# A case report of chronic dacryocystitis caused by nasal stones

**DOI:** 10.1186/s12886-023-03185-9

**Published:** 2023-11-06

**Authors:** Yandi Huo, Luoxiang Li, Ya Mo, Sirui Guo

**Affiliations:** 1 Department of Opthalmology, Chengdu University of Traditional Chinese Medicine, No.37 Shi-Er-Qiao Road, Chengdu, 610072 Sichuan Province People’s Republic of China; 2 Department of Pathology, Hospital of Chengdu University of Traditional Chinese Medicine, No.39 Shi-Er-Qiao Road, Chengdu, 610072 Sichuan Province People’s Republic of China; 3 Department of Opthalmology, Hospital of Chengdu University of Traditional Chinese Medicine, No.39 Shi-Er-Qiao Road, Chengdu, 610072 Sichuan Province People’s Republic of China

**Keywords:** Nasal calculi, Chronic dacryocystitis, Lacrimal CT angiography, Maxillary osteonecrosis, Case report

## Abstract

**Background:**

This paper reports a case of chronic dacryocystitis due to nasal stones.

**Case presentation:**

An 84-year-old male patient was admitted to the hospital with chronic dacryocystitis of the right eye due to tearing and purulent discharge from the right eye for more than 1 month. Antibiotic treatments such as gatifloxacin eye drops were given at other hospitals but did not relieve the symptoms. A computed tomography(CT) scan of the lacrimal duct in our department showed a high-density shadow in the right lacrimal sac area, hypertrophy of the right inferior turbinate, and many nasal calculi in the nasal cavity. The patient was transferred to our otolaryngology department for further treatment, and nasal stones were removed under nasal endoscopy. Three days after surgery, the symptoms affecting the patient's right eye gradually resolved. One month after surgery, the patient underwent a follow-up examination in the ophthalmology clinic; there was no lacrimal purulent discharge from the right eye, and the lacrimal duct could be flushed smoothly.

**Conclusion:**

Chronic dacryocystitis is often caused by primary nasolacrimal duct obstruction. Cases of chronic dacryocystitis caused by secondary nasolacrimal duct obstruction due to nasal stones are rare in the clinic. This case can serve as a reference for the clinical diagnosis and treatment of chronic dacryocystitis.

## Background

Chronic dacryocystitis is a microbial infectious disease secondary to nasolacrimal duct stenosis or obstruction [1]; lacrimal discharge is the main symptom and is usually accompanied by congestion of the conjunctiva of the inner canthus and discharge of mucus or purulent secretions from lacrimal puncta in the compressed area of dacryocystitis. Chronic dacryocystitis is mostly caused by Gram-positive bacteria, mostly *Staphylococcus aureus* [2–4], which can induce serious complications such as keratitis and orbital cellulitis [5, 6]. Rhinitis, inferior turbinate hypertrophy, nasal septum aberrations, nasal polyps, and lacrimal duct trauma are common causes of chronic dacryocystitis. However, chronic dacryocystitis caused by nasal stones is rare in clinical practice. This paper reports a case of chronic dacryocystitis caused by nasal stones.

## Case presentation

**Fig. 1:**
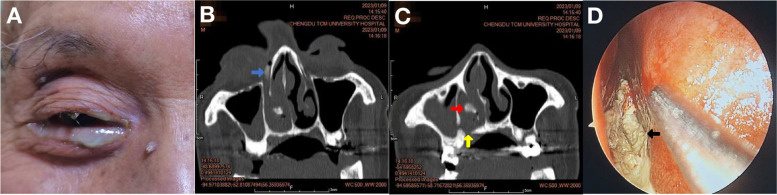
The patient's preoperative eye appearance, CT images of the sinuses, and nasal endoscopic images. **A** The patient showed obvious tearing and purulent discharge in the right eye on admission. **B** Horizontal CT images showed high-density signals of the right dacryocyst and part of the nasolacrimal duct (blue arrowhead). **C** Horizontal CT images showed bone discontinuities in the right mandible (yellow arrowhead) and a soft-tissue-density shadow in the right nasal cavity with a high-density shadow (red arrowhead). **D** nasal endoscopy shows many yellow and white nasal stone-like foreign bodies filled the right common and inferior nasal passages (black arrowhead)

An 84-year-old male patient presented with lacrimal discharge and purulent discharge in his right eye for more than 1 month without any obvious cause. Later, the patient came to our hospital for treatment due to aggravation of the symptoms and was admitted to the hospital with chronic dacryocystitis of the right eye. On admission, the patient showed persistent tearing and purulent discharge from the right eye with a localized cystic bulge in the lacrimal sac area. The patient underwent lacrimal duct irrigation and inserted the needle from the upper lacrimal punctum of the right eye. The flushing liquid flowed back from the lower lacrimal punctum, accompanied by a large amount of purulent secretions. Lacrimal duct CT angiography showed the following a high-density shadow in the right lacrimal sac area and a soft-tissue-density shadow in the right nasal cavity, including a high-density shadow. Nasal endoscopy revealed many yellow‒white nasal calculus-like foreign bodies in the right common and inferior nasal meatus (Fig. 1).

**Fig. 2:**
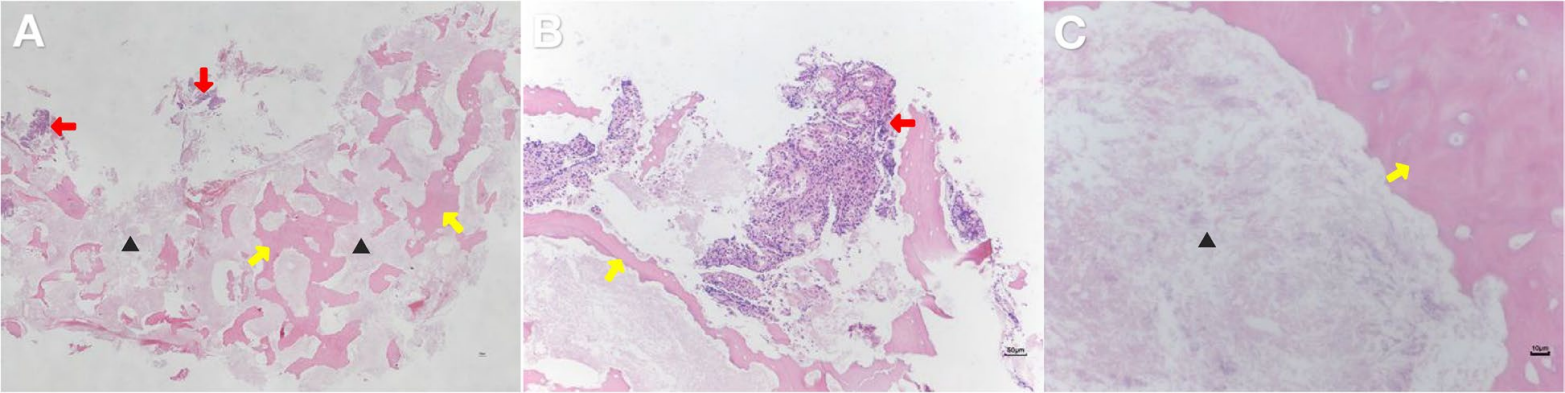
Postoperative pathological images. **A** (panoramic view), **B** (enlarged × 100), and **C** (enlarged × 400) Pathology showed osteocyte degeneration (yellow arrowheads), coagulative necrosis of the interstitium (black triangles), and a small amount of nasal mucosa at the edge of the tissue (red arrowheads)

Inquiring about the patient’s medical history revealed that the patient had received 17 intravenous infusions of zoledronic acid at the other hospital from June 2017 to September 2021 due to severe osteoporosis. In September 2021, the patient was diagnosed at the other hospital with drug-related right maxillary osteonecrosis due to pain and pus in the right maxillary first molar. Then, the zoledronic acid infusions were discontinued, and normal saline was used for daily irrigation. In December 2021, nasal CT at the other hospital showed bone destruction on the right side of the maxilla with soft tissue mass formation and right maxillary sinusitis. Conservative treatment was continued due to the unclear boundary of the bone mass. In June 2022, the patient complained of right nasal obstruction with increased purulent nasal secretion, and in December, he gradually developed chronic dacryocystitis, mainly presenting as lacrimal and purulent discharge from the right eye, and came to our hospital for further diagnosis and treatment. At that time, the patient was transferred to our otolaryngology department for further treatment, and nasal stones were removed under nasal endoscopy. During the operation, it was found that the nasal septum deviated to the right, and there was a large amount of silt-like material in the right inferior meatus and common meatus; the boundary with the surrounding mucosa was clear. Mucosal edema and thickening in the maxillary sinus were found. The pathological results showed the following (D2023-00352): degeneration of bone cells, coagulation necrosis of the stroma, and a small amount of nasal mucosa at the edge of the tissue (Fig. 2). After the operation, ceftizoxime was given for anti-infection therapy, dexamethasone for anti-inflammation therapy, and aminocaproic acid sodium chloride for hemostasis.

**Fig. 3:**
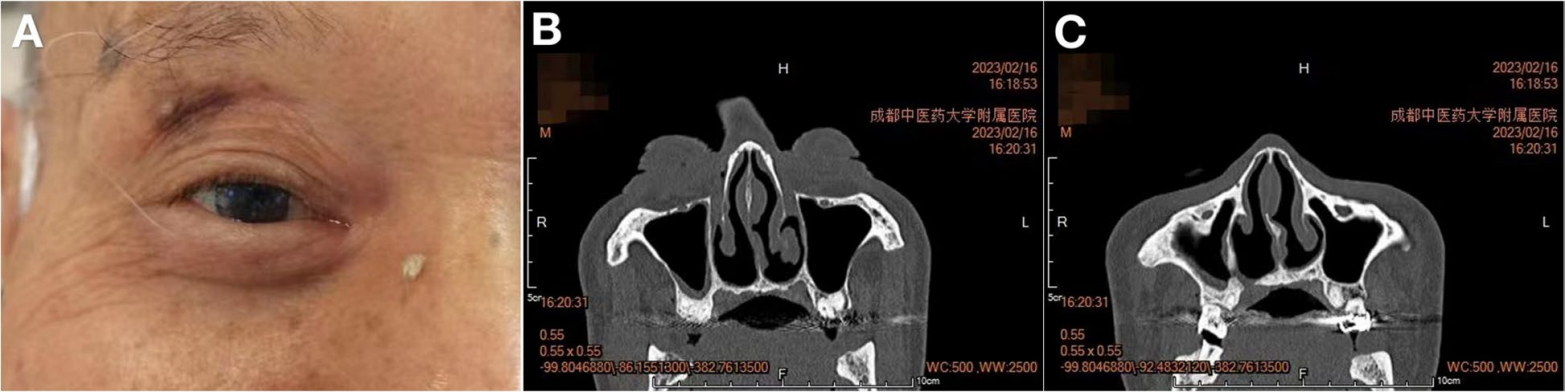
The patient's eye appearance and sinus CT images at 1 month after surgery. **A** The patient showed no symptoms of lacrimation or purulence in the right eye. **B** Horizontal CT images showed that the right sinus and nasolacrimal duct had a uniform density and that the nasolacrimal duct was patent. **C** Horizontal CT images showed no high-density or soft-tissue-density shadows in the right nasal cavity

Three days after surgery, the symptoms affecting the patient's right eye gradually resolved, the lacrimal purulence decreased, and routine blood tests showed the following: white blood cells, 6.06 × 10^9/L (normal range, 3.5–9.5 × 10^9/L); neutrophils, 4.01 × 10^9/L (normal range, 1.8–6.3 × 10^9/L); and lymphocytes, 1.56 × 10^9/L (normal range, 1.1–3.2 × 10^9/L). Other indicators were normal. One month after surgery, the patient underwent a follow-up examination in the ophthalmology clinic; there was no lacrimal purulent discharge from the right eye, and the lacrimal duct could be flushed smoothly. A CT scan of the nasolacrimal duct showed that the right nasolacrimal duct was patent (Fig. 3), and there was no soft-tissue-like shadow or high-density shadow in the right nasal cavity.

## Discussion and conclusions

Nasal calculi are mainly composed of foreign bodies and blood clots in the nasal cavity; calcium phosphate, calcium carbonate, and other tear substances in nasal secretions and tears are deposited around the core, and stones are formed over time. A study by Seyhun et al. found that the most common symptoms of nasal stones were unilateral nasal obstruction and unilateral suppurative rhinorrhea, and between the inferior turbinate and nasal septum was the most common site of stone production (67.7%) [7]. Sinonasal CT examination is the first choice for the diagnosis of rhino lithiasis and usually shows a uniform high-density mass in the center of the lesion with an irregular contour, which can sometimes cause changes such as nasal septum perforations, destruction of the medial wall of the maxillary sinus with recurrent sinusitis, palatal perforations, and oral fistulas [8]. In this case, due to drug-related maxillary osteonecrosis [9] accompanied by soft tissue mass formation and surrounding deposition of inflammatory secretions of the nasal cavity, nasal stones were generated in the inferior nasal meatus, which obstructed the nasolacrimal duct and finally produced chronic dacryocystitis with lacrimal discharge as the main manifestation.

Chronic dacryocystitis is more common in middle-aged and elderly women, and clinical case reports of dacryocystitis in male patients are rare (28.7%, 23.1%, and 32.4%, respectively) [10–12]; additionally, only 1.35% of male patients with dacryocystitis are over 60 years old [12], which may be due to the narrowness of the nasolacrimal duct in the female anatomy [13]. Nasolacrimal duct obstruction is the most important cause of dacryocystitis [14]. Obstruction of the lower lacrimal system causes tear discharge disorders that allow bacteria to multiply, leading to lacrimal sac inflammation, which further aggravates nasolacrimal duct obstruction, creating a vicious cycle [15–17]. An epidemiological survey of dacryocystitis by Khatoon et al. showed that secondary nasolacrimal duct obstruction accounted for approximately 3.78% of cases, much less than primary nasolacrimal duct obstruction (96.23%) [18]. Nasal-related diseases account for approximately 28.6% of dacryocystitis, and the most common causes are deviation of the nasal septum, rhinitis, and hypertrophy of the inferior turbinate on the same side as the infection [14]. Cases related to nasal stones are rarely reported. In clinical practice, chronic dacryocystitis usually has a long onset time, which can be several years. In this case, the patient had right nasal obstruction with increased nasal secretion since June 2022, but the symptoms were ignored because of the long-term pain and purulent discharge in the right posterior maxilla due to maxillary necrosis on the right side.

We report this case because such cases are rare in the clinic, and chronic dacryocystitis caused by nasal calculi can easily be missed.

## Data Availability

The datasets used and/or analyzed during the current study are available from the corresponding author upon reasonable request.

## References

[1] Soriano LM, Damasceno NA, Herzog Neto G, Damasceno EF (2019). Comparative study of the clinical profile of chronic dacryocystitis and chronic rhinosinusitis after external dacryocystorhinostomy. Clin Ophthalmol.

[2] Luo B, Li M, Xiang N, Hu W, Liu R, Yan X (2021). The microbiologic spectrum of dacryocystitis. BMC Ophthalmol.

[3] Chung SY, Rafailov L, Turbin RE, Langer PD (2019). The microbiologic profile of dacryocystitis. Orbit.

[4] Eshraghi B, Abdi P, Akbari M, Fard MA (2014). Microbiologic spectrum of acute and chronic dacryocystitis. Int J Ophthalmol.

[5] Pornpanich K, Luemsamran P, Leelaporn A, Santisuk J, Tesavibul N, Lertsuwanroj B (2016). Microbiology of primary acquired nasolacrimal duct obstruction: simple epiphora, acute dacryocystitis, and chronic dacryocystitis. Clin Ophthalmol.

[6] Nagasato D, Tabuchi H, Yamauchi T, Imamura H, Shimizu Y (2021). Severe corneal melting and perforation secondary to chronic dacryocystitis due to delayed ophthalmology consultation. Oxf Med Case Rep.

[7] Seyhun N, Toprak E, Kaya KS, Dizdar SK, Turgut S (2020). Rhinolithiasis, a rare entity: analysis of 31 cases and literature review. North Clin Istanb.

[8] Lahma J, Hejjouji R, Azzam I, Oujilal A, Essakalli L (2018). Rhinolithiasis: about an observation of a rare condition. Pan Afr Med J.

[9] Limones A, Sáez-Alcaide LM, Díaz-Parreño SA, Helm A, Bornstein MM, Molinero-Mourelle P (2020). Medication-related osteonecrosis of the jaws (MRONJ) in cancer patients treated with denosumab VS. zoledronic acid: A systematic review and meta-analysis. Med Oral Patol Oral Cir Bucal.

[10] Pinar-Sueiro S, Fernández-Hermida RV, Gibelalde A, Martínez-Indart L (2010). Study on the effectiveness of antibiotic prophylaxis in external dacryocystorhinostomy: a review of 697 cases. Ophthalmic Plast Reconstr Surg.

[11] Chen L, Fu T, Gu H, Jie Y, Sun Z, Jiang D (2018). Trends in dacryocystitis in China: A STROBE-compliant article. Medicine (Baltimore).

[12] Badhu B, Dulal S, Kumar S, Thakur SKD, Sood A, Das H (2005). Epidemiology of chronic dacryocystitis and success rate of external dacryocystorhinostomy in Nepal. Orbit.

[13] Baybora H, Uysal HH, Baykal O, Karabela Y (2019). Investigating estrogen and progesterone receptors in the lacrimal sacs of individuals with and without chronic dacryocystitis. Beyoglu Eye J.

[14] Bale RN (1987). Dacryocystitis: bacteriological study and its relation with nasal pathology. Indian J Ophthalmol.

[15] Li Y, Liu X, Zhang W, Song X, Zhang L, Xiao C (2022). Differently expressed circular RNAs in lacrimal sacs from patients with chronic dacryocystitis. Front Genet.

[16] Penttilä E, Smirnov G, Tuomilehto H, Kaarniranta K, Seppä J (2015). Endoscopic dacryocystorhinostomy as treatment for lower lacrimal pathway obstructions in adults: review article. Allergy Rhinol (Providence).

[17] Cnaan RB, Moosajee M, Heatley CJ, Olver JM (2012). Endoscopic endonasal retrieval of a nasolacrimal duct stone via the valve of Hasner in the inferior meatus. Ophthalmic Plast Reconstr Surg.

[18] Khatoon J, Rizvi SAR, Gupta Y, Alam MdS (2021). A prospective study on epidemiology of dacryocystitis at a tertiary eye care center in Northern India. Oman J Ophthalmol.

